# Prospective assessment of vacuum deliveries from midpelvic station in a tertiary care university hospital: Frequency, failure rates, labor characteristics and maternal and neonatal complications

**DOI:** 10.1371/journal.pone.0259926

**Published:** 2021-11-16

**Authors:** Meryam Sugulle, Erna Halldórsdóttir, Janne Kvile, Line Sissel Dahlgaard Berntzen, Anne Flem Jacobsen

**Affiliations:** 1 Division of Gynaecology and Obstetrics, Oslo University Hospital, Oslo, Norway; 2 Institute of Clinical Medicine, Faculty of Medicine, University of Oslo, Oslo, Norway; Mayo Clinic Minnesota, UNITED STATES

## Abstract

**Background:**

Midpelvic vacuum extractions are controversial due to reports of increased risk of maternal and perinatal morbidity and high failure rates. Prospective studies of attempted midpelvic vacuum outcomes are scarce. Our main aims were to assess frequency, failure rates, labor characteristics, maternal and neonatal complications of attempted midpelvic vacuum deliveries, and to compare labor characteristics and complications between successful and failed midpelvic vacuum deliveries.

**Study design:**

Clinical data were obtained prospectively from all attempted vacuum deliveries (n = 891) over a one-year period with a total of 6903 births (overall cesarean section rate 18.2% (n = 1258). Student’s t-test, Mann-Whitney U-test or Chi-square test for group differences were used as appropriate. Odds ratios and 95% confidence intervals are given as indicated. The uni- and multivariable analysis were conducted both as a complete case analysis and with a multiple imputation approach. A p-value of <0.05 was considered statistically significant.

**Results:**

Attempted vacuum extractions from midpelvic station constituted 36.7% (n = 319) of all attempted vacuum extractions (12.9% (n = 891) of all births). Of these 319 midpelvic vacuum extractions, 11.3% (n = 36) failed and final delivery mode was cesarean section in 86.1% (n = 31) and forceps in the remaining 13.9% (n = 5). Successful completion of midpelvic vacuum by 3 pulls or fewer was achieved in 67.1%. There were 3.9% third-degree and no fourth-degree perineal tears. Cup detachments were associated with a significantly increased failure rate (adjusted OR 6.13, 95% CI 2.41–15.56, p< 0.001).

**Conclusion:**

In our study, attempted midpelvic vacuum deliveries had relatively low failure rate, the majority was successfully completed within three pulls and they proved safe to perform as reflected by a low rate of third-degree perineal tears. We provide data for nuanced counseling of women on vacuum extraction as a second stage delivery option in comparable obstetric management settings with relatively high vacuum delivery rates and low cesarean section rates.

## Introduction

Increasing second stage cesarean section (CS) rates and a concomitant decline in operative vaginal deliveries in the United States and European countries [1, 2] warrant increased clinical and academic focus on mode of delivery in second stage of labor. Both operative vaginal delivery (OVD) rates and their failure rates vary internationally, ranging from 4.6% [3] to 20% [3–6], and 4.6% to 30.1% [4, 5, 7–9], respectively, depending on the obstetric population and instrument studied. Midpelvic OVD is of special debate, since, compared to CS, increased risk of maternal and perinatal morbidity and mortality has been shown in recent register-based studies [10–12]. Further, both high failure rates of up to 15% and high rates of third- and fourth- degree perineal tears of up to 10.3% [11] have been reported for midpelvic vacuum extractions (VE). Studies prospectively describing attempted midpelvic VE outcomes are scarce and include multi-center studies comprising obstetric units with different obstetric management guidelines [6] or studies of several modalities of OVD, where VE is only one of the lesser applied options [3].

The recent, large, prospective study by Ducarme et al., including attempted midpelvic OVD assisted by either VE, forceps or Thierry’s spatula in singleton term pregnancies, showed no increased risk for severe short term maternal or neonatal morbidity in OVD from midpelvic compared to low or outlet station [3].

Obstetric practice in Norway is characterized by relatively low overall CS rates (approximately 16%) and relatively high OVD rates (approximately 11%) in international comparison, with s rates during the last decade [13].

Our main aim was to assess the frequency, failure rates, labor characteristics, maternal and fetal complications of attempted midpelvic VE. Our secondary aim was to compare labor characteristics and complications between successful and failed midpelvic VE.

## Materials and methods

### Study population and data registration

All women who underwent any attempt of VE at Oslo University Hospital, Ullevål from October 2014 until October 2015 were included in this prospective, observational study. Our high volume tertiary care obstetric unit has over the past decade (years 2010–2019) had relatively stable annual rates of VE (range 10.3–12.3%) and low overall CS rates (range 14.1–20.3%) [13]. Attempted VE was defined vacuum cup placement with the intent to deliver the baby, regardless of success. Indication for VE was not influenced by the ongoing study. VE were performed by either the obstetric resident and/or the specialist consultant. All women scheduled for delivery during the study period had received study information prior to delivery. This quality surveillance study was approved by the Oslo University Hospital Data Protection Authority, and required passive informed consent.

At delivery, the attending delivery nurse/midwife recorded cup size, the number of cup detachments or deliberate repositioning, the number of pulls, the time for start and stop of VE and birth, and final delivery mode on a pro forma. The operator detailed indication for delivery, fetal pelvic station and position, cup placement and degree of molding directly after the attempted VE (S1 File). Neonatal Apgar score at one, five and ten minutes as well as maternal blood loss was retrieved from the electronic obstetric patient records, and all records of all VE’s were reviewed by the researcher (MS). Gestational age was recorded in complete weeks based on routine ultrasound dating at gestational week 18.

### Definitions

A failed VE was defined as a delivery requiring sequential use of forceps or if the final delivery mode was CS.

A midpelvic VE was defined as a VE where the fetal head was engaged in the pelvis with the leading part of the skull at or below the level of the ischial spines but above station + 2 cm, according to national guidelines [14].

The choice of instrument, i.e. either the metal cup of the Bird type (50 mm or 60 mm diameter) or the Kiwi OmniCup, as well as the decision for a mediolateral episiotomy were left to the operators’ discretion.

The time from vacuum application to each cup detachment or deliberate removal, or delivery was measured in completed minutes. Duration of VE was defined as the total amount of these time measures.

Operator category was defined as either ‘Resident’, ‘Specialist consultant ≤5 years’ and ‘Specialist consultant >5 years’. Time point for Norwegian Board Certification in Obstetrics and Gynaecology was used for category assignment.

Shoulder dystocia was defined according to national guidelines as either the need for any additional maneuver to obtain the expulsion of the shoulders apart from gentle traction or as lack of expulsion of the shoulders within the next contraction after the birth of the baby’s head [15].

Perineal tears were classified according to national guidelines [16] which correspond to the Royal College of Obstetricians and Gynaecologists (RCOG) definitions [17]. Thus, a third-degree perineal tear involves the anal sphincter complex, with further subdivision into grade 3a, b, c depending on the extent of external anal sphincter (EAS) affection and involvement of the internal anal sphincter (IAS). A fourth-degree tear affects EAS and IAS and anorectal mucosa [16].

Postpartum hemorrhage (PPH) was defined as blood loss greater than 500 mL within the first 24 hours after delivery, and severe PPH as a blood loss of greater than 1500 mL, both according to national guidelines [18].

### Statistical analyses

Continuous data are presented as means and standard deviation or medians and interquartile range (IQR) and categorical data are presented as percentages. Group differences were tested with Student’s t-test or Mann-Whitney U-test for continuous variables and with Chi-square test for proportions. A p-value of <0.05 was considered statistically significant. The uni- and multivariable analysis were conducted as a complete case analysis; as well as with a multiple imputation approach, (5 times) for variables with missing values. Multivariable linear regression analysis was used to adjust for gestational age, birth weight and suspicion of malposition. SPSS version 25.0 (IBM Corp. Armonk, NY, USA) was used for analyses.

### Ethics

This quality surveillance study was approved by the Oslo University Hospital Data Protection Authority (Reference 2014/10362), and required passive informed consent.

## Results

### Delivery outcome

**Fig 1:**
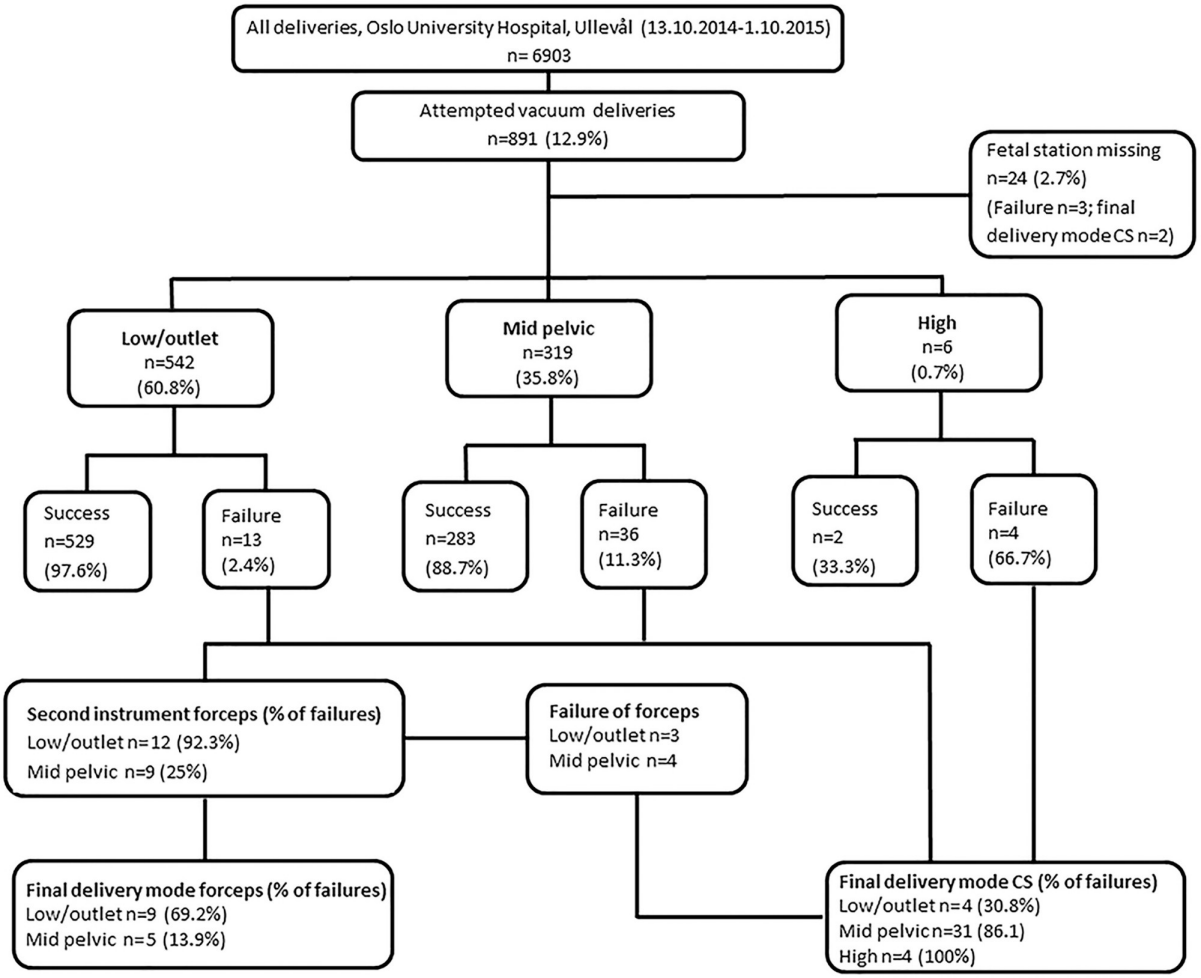
Study inclusion flow chart and outcome of attempted VE for the total cohort according to fetal station. CS, Cesarean section.

During the study period there were 6903 deliveries, thereof 12.2% (n = 840) acute CS and an overall total CS rate of 18.2% (n = 1258). Attempted VE was recorded in 12.9% (n = 891) deliveries. The outcome of all attempted VE according to fetal station is shown in Fig 1. There were no missing data for the primary outcome “failed VE”. Due to missing information on fetal station 2.7% (24/891) of the VE were excluded from further analyses. Attempted VE from midpelvic station occurred in 35.8% (319/891) of all VE with a failure rate of 11.3% (36/319) (Fig 1).

Maternal and neonatal characteristics in attempted midpelvic VE categorized according to success or failure are shown in Table 1. There were no significant differences between the two groups except for a higher median gestational age at delivery in the failure group.

**Table 1:** Maternal and neonatal characteristics in attempted midpelvic vacuum extractions (n = 319) categorized according to success or failure.

Characteristics	Successful midpelvic VE (n = 283)	Failed midpelvic VE (n = 36)	P-value
Maternal age in years, mean (SD)	31.9 (±4,8)	32.4 (±4.7)	0.547
Nulliparous, n (%)	229 (80.9)	30 (83.3)	0.727
Prior vaginal delivery*	18 (33.3)	3 (50)	
Prior CS, n (%)**	20 (37.0)	3 (50)	0.858
Missing, n (%)	16 (29.6)	0	-
Singleton, n (%)	271 (95.8)	36 (100)	0.208
Twins, n (%)	12	0	-
Twin A	6		
Twin B	6		
Fetal sex, n (% male)	164 (58)	19 (52.8)	0.554
GA at delivery in weeks, median (IQR)	40.6 (39.6–41.3)	41.4 (40.42)	0.015
Newborn weight in grams, mean (SD)	3571 (±548)	3695 (±470)	0.198
Missing, n (%)	16 (5.7)	0 (0)	-

CS, Cesarean section; GA, Gestational age; IQR, Interquartile range; SD, Standard Deviation; VE, Vacuum extraction.

*At least on prior vaginal delivery in multiparous women.

**Only prior CS, no vaginal deliveries in any other pregnancies.

### Labor characteristics

Labor characteristics for the two midpelvic VE groups are shown in Table 2.

**Table 2:** Labor characteristics and operator category in attempted midpelvic vacuum extractions (n = 319) with odds ratio indicating the risk of failure.

Characteristics	Successful midpelvic VE (n = 283) n (%)	Failed midpelvic VE (n = 36) n (%)	Crude OR (95% CI)	P-value
Spontaneous labor onset (ref)	194 (68.5)	24 (66.7)		
Induction of labor	89 (31.5)	12 (33.3)	1.09 (0.52–2.27)	0.819
Local anesthesia/pudendal block/none (ref)	71 (25.1)	10 (27.8)		
Epidural	200 (70.7)	22 (61.1)	0.79 (0.36–1.75)	0.564
Missing	12 (4.2)	4 (11.1)		
Indication for delivery				
“Fetal distress” (ref)	172 (60.8)	21(58.3)		
“Arrested labor”	111(39.2)	15 (41.7)	1.10 (0.55–2.24)	0.778
Occiput anterior (ref)	151 (53.4)	16 (44.4)		
Suspicion of malposition (OP or OT)	57 (20.1)	12 (42.9)	1.99 (0.89–4.46)	0.096
Diagnosis not possible	17 (6.0)	1 (2.8)		
Missing	58 (20.5)	7 (19.4)		
Birthweight				
<4000g (ref)	201 (71.0)	26 (72.2)		
≥4000g	66 (23.3)	10 (27.8)	1.17 (0.54–2.55)	0.691
Missing	16 (5.7)	0		
Vacuum duration				
≤ 7 minutes (ref)	148 (52.3)	10 (27.8)		
>7 minutes	114 (40.3)	22 (61.1)	2.86 (1.30–6.27)	0.009
Missing	21 (7.4)	4 (11.1)		
Cup size (final)				
50 mm (ref)	164 (57.9)	21 (70.0)		
60 mm	100 (35.3)	9 (30.0)	0.70 (0.31–1.59)	0.399
KiWi OmniCup	5 (1.9)	0		
Missing,	14 (4.9)	6		
Total number of pulls				
≤3 (ref)	190 (67.1)	21 (58.3)		
>3	89 (31.5)	13 (36.1)	1.32 (0.63–2.76)	0.458
Missing, n (%)	4 (1.4)	2 (5.6)		
Cup detachments				
None (ref)	253 (89.4)	18 (50.0)		
≤2	27 (9.5)	15 (41.7)	7.81 (3.54–17.24)	≤0.001
Missing	3 (1.1)	3 (8.3)		
First operator category				
Resident (ref)	196 (69.3)	23 (63.9)		
Specialist consultant ≤5 yrs	50 (17.7)	6 (16.7)	1.01 (0.39–2.63)	0.972
Specialist consultant >5 yrs	37 (13.1)	7 (19.4)	1.60 (0.64–4.0)	0.312

CI, Confidence interval; Ref, reference category; OP, Occiput posterior; OR, Odds ratio; OT, Occiput transverse; VE, Vacuum extraction.

In univariable analyses, a non-significant trend towards higher failure rate in cases of suspicion of malposition (either occiput posterior or occiput transverse) was seen (Table 2). Median duration of midpelvic VE was 7 minutes (IQR 5–11), and a duration above 7 minutes was associated with a significantly higher failure risk in univariable analysis (OR 2.86,95% CI 1.3–6.3; p = 0.009), but not in multivariable analysis after adjustment for gestational age, birth weight, suspicion of malposition and cup detachments (Table 3).

**Table 3:** Multivariable linear regression analysis for attempted midpelvic vacuum extractions (n = 319) with odds ratio indicating the risk of failure.

Characteristics	Adjusted OR*	P-value*	Adjusted OR**	P-value**
Cup detachments				
None (ref)	1		1	
≤2	6.13 (2.41–15.56)	≤ 0.001	7.22 (2.61–19.97)	≤ 0.001
Vacuum duration				
≤ 7 minutes (ref)	1		1	
>7 minutes	1.29 (0.48–3.45)	0.617	1.27 (0.49–3.25)	0.623

Ref, reference category; OR, Odds ratio; OT, Occiput transverse.

*Adjustment for gestational age, birth weight and suspicion of malposition for cup detachments, and in addition for cup detachments in vacuum duration by forced entry.

** Adjustment for confounders as above, after multiple imputation (5 times).

Overall, residents commenced attempted midpelvic VE in 68.7% (219/319) and in 48.9% (156/319) two or more operators were present. Active involvement of the second operator (specialist consultant) as documented by executed pulls occurred in 28.2% (44/156) of these deliveries. Successful completion of midpelvic VE by 6 pulls occurred in 96.8% (274/283) and 3 pulls or fewer in in 67.1% (Table 2). There was no significant association between number of pulls and midpelvic VE failure (Table 2).

Cup detachment occurred in a total of 13.2% (42/319) of the attempted midpelvic VE, thereof one detachment in 78.6% (33/42), the remainder with a maximum of two detachments. Failure risk was significantly increased in those with any cup detachment compared to those without, in both uni- and multivariable analyses (OR = 7.81, 95% CI 3.54–17.2, and OR 6.13, 95% CI 2.41–15.56, respectively, both p< 0.001, Table 3).

### Maternal and neonatal complications

Third-degree perineal tears occurred in 3.9% (11/283) of the successfully completed midpelvic VE (Table 4, S1 Table). There were no fourth-degree perineal tears. Episiotomy was done in 74.5% (212/283) of the successful and in 36.1% (13/36) of the failed midpelvic VE. Of the latter, two women eventually delivered vaginally. Data on episiotomy were missing in 20.8% (59/283) and 25% (9/36), respectively.

**Table 4:** Maternal outcome in attempted midpelvic vacuum extractions (n = 319) categorized according to success or failure.

Maternal outcome	Successful midpelvic VE (n = 283) n (%)	Failed midpelvic VE (n = 36) n (%)	P-value
Cesarean delivery	-	31 (86.1)	
Unintended uterine incision extension	-	11 (35.5)	
Third-degree perineal tear	11 (3.9)	-	
PPH			
>500 mL	73 (25.8)	15 (41.7)	0.014
>1000 mL	24 (8.5)	6 (16.7)	0.027
Missing	15 (5.3)	2 (5.5)	

PPH, Post-partum hemorrhage; VE, Vacuum extraction.

Postpartum hemorrhage (PPH) complicated 37.0% (118/319) midpelvic VE, thereof 9.4% (30/319) with severe PPH (blood loss greater than 1000 mL). Data on blood loss were missing in 5.3% (17/319) deliveries. Occurrence of PPH and severe PPH was significantly associated with VE failure in univariable analyses (PPH: OR = 2.7, 95% CI 1.23–5.97, p = 0.014; severe PPH: OR 3.29, 95% CI 1.14–9.47, p = 0.027).

**Fig 2:**
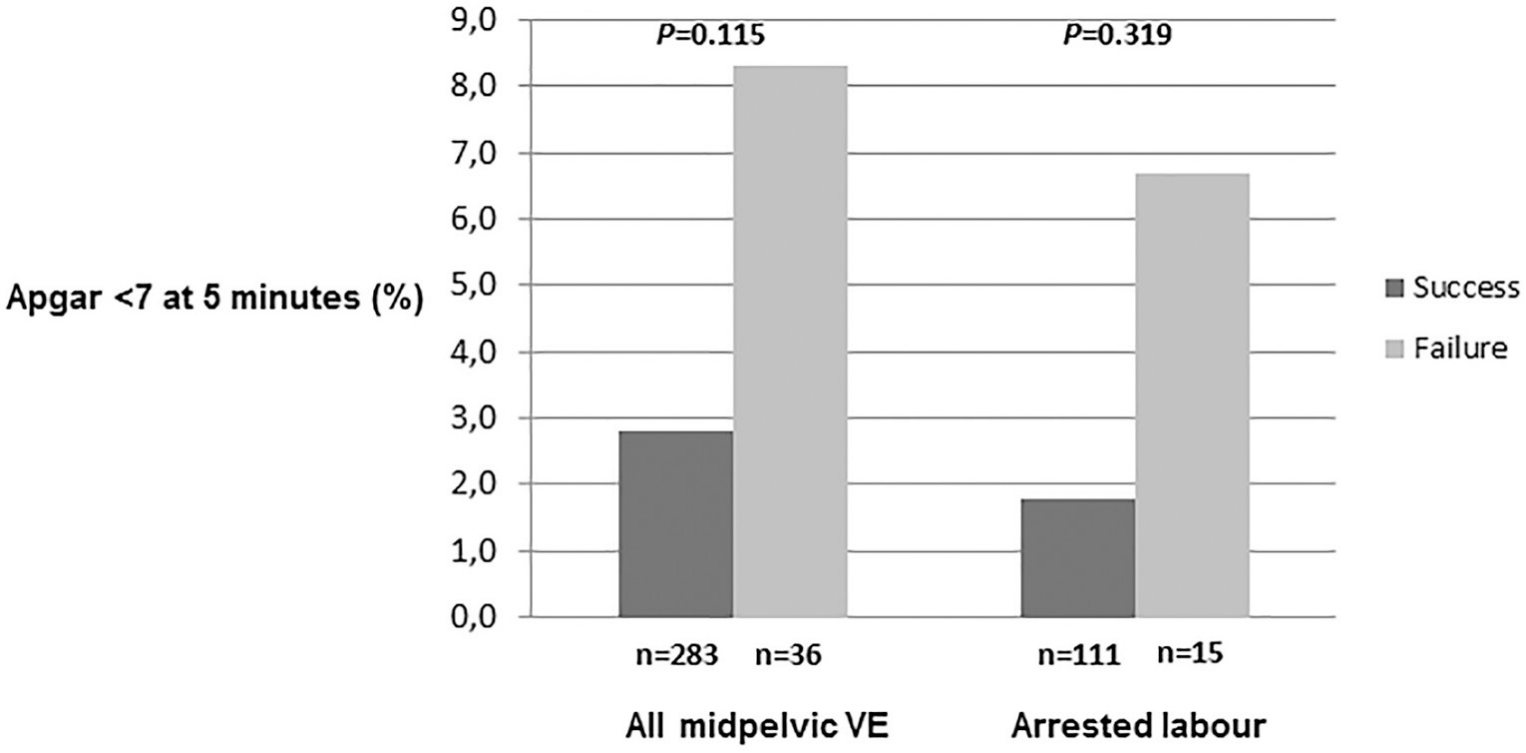
Proportion of neonates with Apgar <7 at 5 minutes (%) according to success or failure of attempted midpelvic vacuum and delivery indication “arrested labour”. VE, Vacuum extractions. Multiple imputation did not change the significance of any results (Table 3 and S2 Table).

Data on Apgar score at 1 and 5 minutes were missing in 5 deliveries, none failed. In the total group of midpelvic VE, 3.4% (11/319) of the neonates had an Apgar score < 7 at 5 minutes. There were no significant associations between rates of Apgar score <7 at 5 minutes and midpelvic VE failures regardless of indication for delivery (Fig 2). Shoulder dystocia occurred in 9.9% (28/283) of successful midpelvic VE.

There were no neonatal or maternal deaths.

## Discussion

### Main findings

To the best of our knowledge, this is the largest prospective, single center study of midpelvic VE in a general obstetric population in a setting of a relatively high overall VE rate and concomitant low VE failure rate in international comparison [7, 19, 20].

Our main findings are firstly, a higher rate of midpelvic VE and a lower failure rate than register based studies indicate. Secondly, the frequency of third-degree perineal tears was substantially lower compared to earlier reports, and there were no fourth-degree tears. Thirdly, successful completion of midpelvic vacuum was achieved by three pulls or fewer in the majority of deliveries. Lastly, cup detachments were significantly associated with an increased midpelvic VE failure rate, also after adjustment for preselected potential confounders in multivariable analyses.

### Interpretation

Studies prospectively describing the obstetric characteristics and outcomes of attempted midpelvic VE are scarce. Ducarme et al’s study included nearly 400 midpelvic OVD, of which 38 were midpelvic VE, the remainder consisting of forceps or Thierry’s spatula [3]. Ahlberg et al. followed 596 VE of which 450 (75%) were classified as midpelvic, however their study included 6 different obstetric units of which only three supplied guidelines containing information about VE classification by station [6]. In our study, data on fetal station was collected from both the delivery pro forma and electronic obstetric patient records.

Compared to studies from Canada and the UK with rates of 20 and 26%, respectively [10, 11, 21], our rate of attempted midpelvic VE was higher, and the failure rate was lower [10, 11]. We could not identify data from prospective studies specifying failure rates for attempted midpelvic VE.

Our important finding of the absence of fourth-degree perineal tears and the relatively low frequency of third-degree perineal tears corresponds with low rates of third- or fourth-degree perineal tears of 2.1% in midpelvic OVD reported by Ducarme et al. [3]. Both ours and Ducarme’s results differ substantially from previously reported rates of third- or fourth-degree perineal tears of 10.9% in a prospective series of midpelvic rotational VE [21] and from register-based data yielding rates of 8.5–16.2% in attempted midpelvic VE [10–12]. Muraca et al. strongly advocated that women should be informed about the substantially increased risk of anal sphincter trauma after midpelvic OVD [11, 12]. Our results convincingly show that severe maternal trauma in terms of high grade perineal tears is less frequent than studies from different obstetric settings indicate. At our department, we provide continuous systematic perineal protection skills training for all physicians and midwives [22].

Episiotomy is recommended in all OVD in nulliparous women in our national guidelines [14]. Of concern are the unnecessary episiotomies in 1/3 of the women with failed midpelvic VE who did not deliver vaginally. For failed VE from any station, an episiotomy frequency of 19% has been reported [5], but published data for failed midpelvic VE are lacking.

Postpartum hemorrhage plays an important role for maternal morbidity, with the potential for blood transfusion requirement, protracted reconvalescence postpartum and maternal death in the most severe cases [23]. Several ante- and intrapartum risk factors for PPH are known, and both instrumental vaginal delivery, elective and intrapartum cesarean section are among the latter [24]. Our finding of an increased risk for postpartum hemorrhage in failed midpelvic VE compared to successful VE underscores the importance of identification of antenatal PPH risk factors as well as intrapartum prophylactic measures in every woman booked for delivery in order to optimize maternal health.

A 2.6 fold increased risk of Apgar score < 7 at 5 minutes in failed compared to successful VE, adjusted for fetal station and indication for delivery has been reported [5]. We did not find any significant association between midpelvic VE failure and Apgar score <7 at 5 minutes regardless of indication for delivery. This is most likely due to lack of statistical power for the outcome. The higher frequency of shoulder dystocia in successful midpelvic VE compared to other studies [21, 25] may partly be due to a broader definition of shoulder dystocia in our national [15] as opposed to RCOG or American College of Obstetricians and Gynecologists guidelines [26, 27].

Published data on the average number of pulls in midpelvic VE are lacking. In our study, almost all successfully completed midpelvic VE were achieved by six pulls or fewer, and the majority by three pulls or fewer, thus fulfilling the recommended completion within three pulls [28]. Corresponding to findings for vacuum failures from any station, we observed a higher proportion of a total number of pulls exceeding three in midpelvic VE failures compared to successful ones [4], however this difference was not statistically significant. Increased risk of neonatal trauma in failed OVD with more than three pulls justifies the recommended upper limit of 3 pulls, with possible exception where descent with each pull is clearly recorded [29]. The Norwegian guideline does not give a maximum number of pulls, but requires descent upon the first three pulls and a completion of delivery within 20 minutes [14]. The median extraction time of 7 minutes in our study was comparable to a median time of 6 minutes observed in an earlier study of mainly midpelvic VE [6]. After adjustment for number of cup detachments, gestational age, birth weight and suspicion of malposition, the association with increased risk of failure in VE exceeding seven minutes was not sustained.

Our cup detachment rate of 13.2% in attempted midpelvic VE is lower compared to earlier studies reporting any detachment in 15.6% of all extractions including predominantly midpelvic VE [6, 20]. The Norwegian guideline advises to abandon the VE attempt after three cup detachments, if not indicated earlier. The fact that there were no midpelvic VE attempt with three cup detachments and only few deliveries with two detachments indicates guideline adherence. Cup detachment has been associated with suboptimal cup placement and traction [30]. Thus, the association of cup detachment with an up to seven times increased risk for midpelvic VE failure sustained in multivariable analyses is in line with these considerations.

### Study strengths and limitations

The main strength of our study is the prospective design and its setting in a single, high volume tertiary university hospital with low CS and high OVD rates that have been stable over the last decade. The exclusive focus on the vacuum extractor as instrument and the use of one predominantly cup type (metal) grants homogeneity. The restriction of the study period to one year secures that obstetric staff changes are kept to a minimum.

A limitation is the lack of maternal height and weight, and the incomplete data on prior CS. However, only 1/5 of the women were multiparous, and prior CS would have to be differentiated into first and second stage CS, rendering small groups for statistical analyses. Maternal blood loss is in most deliveries estimated visually, and only in suspicion of more severe bleeding assessed by weighing or intraoperatively by collector bags.

Neonatal outcome measures were limited, i.e. data on more common complications such as scalp lacerations, cephalhematomas as well as subgaleal hematomas and rare events such as intracranial hemorrhage or retinal hemorrhage were not available.

## Conclusion

In our high volume obstetric unit, attempted midpelvic vacuum deliveries had relatively low failure rate, the majority was successfully completed within three pulls and they proved safe to perform as reflected by a low rate of third-degree and an absence of fourth-degree perineal tears. Our findings provide data for nuanced counseling of women on second stage delivery options in comparable obstetric management settings as ours, characterized by relatively high OVD rates and low CS rates.

## Supporting information

S1 File Delivery pro forma. (PDF)

S1 Table Details on indication for delivery and operator category in successful midpelvic VE complicated with third-degree perineal tears. NA, not applicable; yrs, years.
(DOCX)

S2 Table Details of labor and operator grade according to success for midpelvic VE (n = 319) with odds ratio indicating the risk of failure after multiple imputation (5 times) for clinical variables with missing data. CI, Confidence interval; Ref, reference category; OP, Occiput posterior; OR, Odds ratio; OT, Occiput transverse.
(DOCX)
