# Supplementary material for: Prospective assessment of vacuum deliveries from midpelvic station in a tertiary care university hospital: Frequency, failure rates, labor characteristics and maternal and neonatal complications
Source: PLoS One. 2021 Nov 16;16(11):e0259926. doi: 10.1371/journal.pone.0259926 (PMC8594828; doi:10.1371/journal.pone.0259926)
Supplement: S1 File — (PDF) [file pone.0259926.s001.pdf]

OPERATØRSKJEMA VAKUUMFORLØSNING

Pasientens klistrelapp

- Dato:____________ Lege1: _____________ Lege 2:____________
- **INDIKASJON:** ☐ Patologisk CTG/laktat ☐ Protrahert stadium II

  ☐ Annet, spesifiser_____________________________

- **HODETS HØYDESTAND I RELASJON TIL SPINAE ISCHIADICAE:**

UNDER ______________________________________ **VED**

☐+1 ☐ 0

☐+2

☐+3

☐+4

☐+5

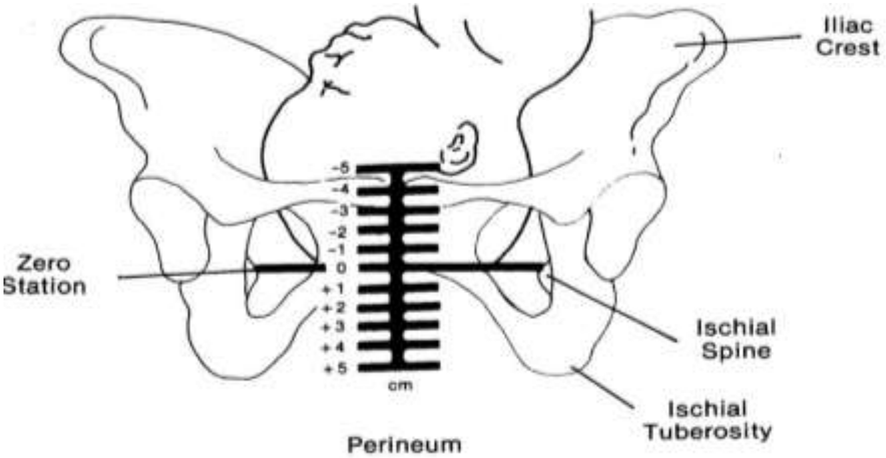

- **TEGN INN HODET MED <u>FONTANELLER OG PILSØM</u> VED VAKUUMANLEGGELSE:**

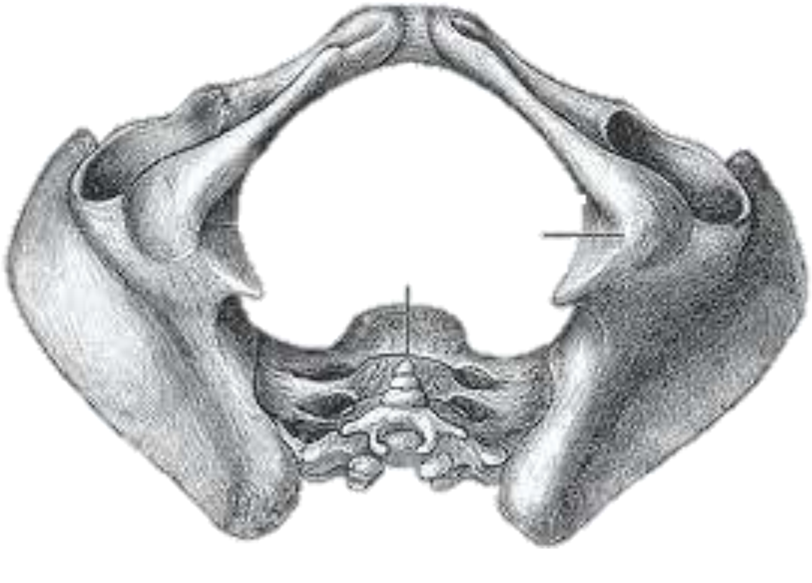

SNU ARKET!* SNU ARKET!* SNU ARKET!*

- **TEGN INN HVOR DU PLASSERTE KOPPEN:**

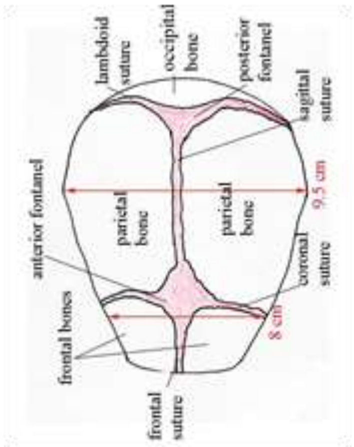

- **FORELÅ DET MOULDING/OVERRIDENDE SUTURER?**

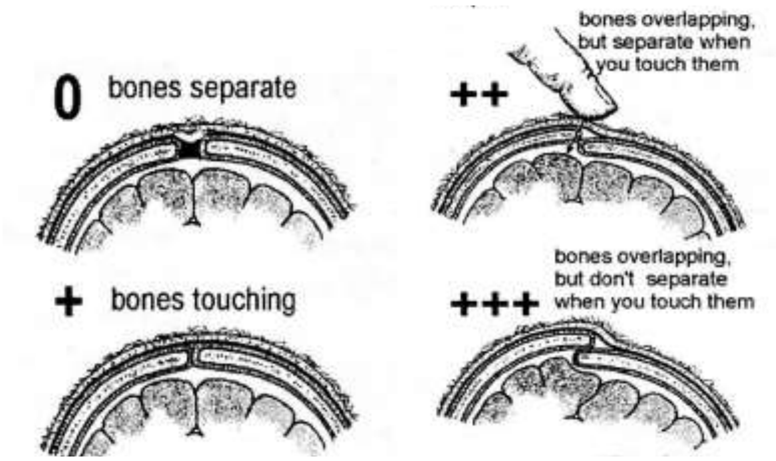

☐ 0

☐ +

☐ ++

☐ +++

- Kopp nr: 5☐ 6☐ KiWi☐
- Koppglipp: nei☐ ja☐;antall: 1☐ 2☐ 3☐
- Kopp tatt av for korrektur av plassering? nei☐ ja☐;antall: 1☐ 2☐ 3☐
- Antall drag lege 1: 1☐ 2☐ 3☐ 4☐ 5☐ 6☐
- Antall drag lege 2: 1☐ 2☐ 3☐ 4☐ 5☐ 6☐

- Start vakuum(forsøk) klokken: ________________
- Slutt vakuum(forsøk) klokken: ________________
- Endelig forløsningsmetode: Vakuum ☐ Tang ☐

Sectio grad 1 ☐ grad 2 ☐

<u>TAKK! Skjema legges i skuff merket «vakuumforløsninger» på vaktrommet.</u>
