# Supplementary material for: Prospective assessment of vacuum deliveries from midpelvic station in a tertiary care university hospital: Frequency, failure rates, labor characteristics and maternal and neonatal complications
Source: PLoS One. 2021 Nov 16;16(11):e0259926. doi: 10.1371/journal.pone.0259926 (PMC8594828; doi:10.1371/journal.pone.0259926)
Supplement: S1 Table — NA, not applicable; yrs, years. (DOCX) [file pone.0259926.s002.docx]

**S1 Table. Details on indication for delivery and operator category in mipelvic VE complicated with third-degree perineal tears**

| Grade of perineal tear [1, 2] | Indication for delivery | First operator category | Supervised by specialist consultant |
| --- | --- | --- | --- |
| 3 A | Fetal distress | Specialist consultant ≤5 yrs | NA |
| 3 A | Arrested labour | Resident | no |
| 3 A | Fetal distress | Resident | no |
| 3 A | Arrested labour | Specialist consultant >5 yrs | NA |
| 3 A | Fetal distress | Resident | yes |
| 3 B | Arrested labour | Resident | yes |
| 3 B | Fetal distress | Resident | yes |
| 3 B | Fetal distress | Resident | no |
| 3 B | Fetal distress | Specialist consultant >5 yrs | NA |
| 3 B | Arrested labour | Specialist consultant ≤5 yrs | NA |
| 3 B | Arrested labour | Resident | yes |

NA, Not applicable; yrs,years

1. Laine K, Spydslaug A, Baghestan E, Norderval S, Olsen I, Fodstad K. Perinealskade og anal sfinkterskade ved fødsel: Norsk gynekologisk forening. Veileder i fødselshjelp (2020); [updated 14.10.2020. 17.02.2020:[Available from: <https://www.legeforeningen.no/foreningsledd/fagmed/norsk-gynekologisk-forening/veiledere/veileder-i-fodselshjelp-2020/>.]

2. Third- and Fourth-degree Perineal Tears, Management (Green-top Guideline No. 29): RCOG; [Available from: <https://www.rcog.org.uk/en/guidelines-research-services/guidelines/gtg29/>.]
