# Supplementary material for: Prospective assessment of vacuum deliveries from midpelvic station in a tertiary care university hospital: Frequency, failure rates, labor characteristics and maternal and neonatal complications
Source: PLoS One. 2021 Nov 16;16(11):e0259926. doi: 10.1371/journal.pone.0259926 (PMC8594828; doi:10.1371/journal.pone.0259926)
Supplement: S2 Table — CI, Confidence interval; Ref, reference category; OP, Occiput posterior; OR, Odds ratio; OT, Occiput transverse. (DOCX) [file pone.0259926.s003.docx]

S2 Table. Details of labor and operator grade according to success for midpelvic vacuum extractions (n=319) with odds ratio indicating the risk of failure after multiple imputation (5 times) for clinical variables with missing data

| Characteristics | Successful midpelvic VE  (n=283)  n (%) | Failed midpelvic VE  (n=36)  n (%) | Crude OR (95% CI) | P-value |
| --- | --- | --- | --- | --- |
| Local anesthesia/pudendal block/none (ref)  Epidural  Missing, n (%) | 71 (25.1)  200 (70.7)  12 (4.2) | 10 (27.8)  22 (61.1)  4 (11.1) | 0.87 (0.39-1.91) | 0.724 |
| Occiput anterior (ref)  Suspicion of malposition (OP or OT)  Diagnosis not possible  Missing, n (%) | 151 (53.4)  57 (20.1)  17 (6.0)  58 (20.5) | 16 (44.4)  12 (42.9)  1 (2.8)  7 (19.4) | 0.98 ( 0.79- 1.22) | 0.875 |
| Birthweight  <4000g (ref)  ≥4000g  Missing, n (%) | 201 (71.0)  66 (23.3)  16 (5.7) | 26 (72.2)  10 (27.8)  0 | 1.20 (0.55-2.62) | 0.644 |
| Cup size (final)  50 mm (ref)  60 mm  KiWi OmniCup  Missing, n (%) | 164 (57.9)  100 (35.3)  5 (1.9)  14 (4.9) | 21 (70.0)  9 (30.0)  0  6 | 0.75 (0.35-1.63) | 0.470 |
| Total number of pulls  ≤3 (ref)  >3  Missing, n (%) | 190 (67.1)  89 (31.5)  4 (1.4) | 21 (58.3)  13 (36.1)  2 (5.6) | 1.32 (0.63-2.76) | 0.458 |

CI, Confidence interval; Ref, reference category; OP, Occiput posterior; OR, Odds ratio; OT, Occiput transverse; VE, Vacuum extraction
